# Admission NT-ProBNP in Myocardial Infarction: an Alert Sign?

**DOI:** 10.36660/abc.20190685

**Published:** 2019-12

**Authors:** Luís Beck da Silva

**Affiliations:** Hospital de Clínicas de Porto Alegre (HCPA), Porto Alegre, RS - Brazil

**Keywords:** Myocardial Infarction, Heart Failure, Ventricular Dysfunction, Left, Natriuretic Peptides/metabolismo, Natriuretic Peptide Brain/metabolism, Echocardiography/diagnostic imaging, Magnetic Resonance Spectroscopy/methods.

This issue of *Arquivos Brasileiros de Cardiologia *brings a paper entitled “The Usefulness of Admission Plasma NT-pro BNP Level to Predict Left Ventricular Aneurysm Formation after Acute ST-Segment Elevation Myocardial Infarction”.^1^ The authors bring a cohort of 1,519 post-acute ST-segment elevation myocardial infarction (STEMI) who were followed-uP for at least six months. Despite its observational and retrograde design, the authors were straightforward in looking for predictive variables that could foresee the occurrence of left ventricular aneurysms (LVA). Among other major clinical aspects such as previous coronary artery bypass graft, post-MI heart failure, younger age, smoking and no-reflow phenomenon; authors highlighted the importance of high NT-proBNP at admission as a predictor of LVA formation after acute STEMI.

I would probably highlight one weakness and a potentially positive aspect of their work.

The weakness is that a LVA will never be diagnosed by a NT-Pro-BNP level and will always be found, confirmed and/or followed by an image test (Echo, CMR, etc.). NT-ProBNP usually and reliably identifies patients who are sicker or more congested, either in acute,^2^ or in chronic heart failure,^3^ or even without heart failure.^4^

The potentially positive one was, interestingly, what the authors have considered their limitation: that the NT-ProBNP values have been collected at admission. Having a high natriuretic peptide level at the admission of a STEMI patient could be a predictive variable of a clinical event, such as LVA formation, in six months. It was there, on the “Limitations” section, the best and most clinically relevant information.
