# Erratum

**DOI:** 10.36660/abc.20200088

**Published:** 2020-02

**December 2019 Issue, vol.113 (6), page 1138**

In Short Editrorial “Admission NT-ProBNP in Myocardial Infarction: an Alert Sign?”, consider Luís Beck-da-Silva as the correct form for the name of the author Luís Beck da Silva.

